# Factors influencing reselection of village doctors in rural-oriented tuition-waived medical education program in Shanghai, China: a cross-sectional study

**DOI:** 10.3389/fpubh.2024.1511709

**Published:** 2025-01-20

**Authors:** Jing Gao, Juhua Zhang, Chenlei Shi, Aiqin Fan, Peng Zhang

**Affiliations:** ^1^School of Clinical Medicine, Shanghai University of Medicine and Health Sciences, Shanghai, China; ^2^Shanghai University of Medicine and Health Sciences Affiliated Zhoupu Hospital, Shanghai, China; ^3^Shanghai Pudong New Area Sunqiao Community Health Service Center, Shanghai, China; ^4^School of Management, Hainan Medical University, Haikou, China

**Keywords:** new generation of village doctors, questionnaire survey, rural-oriented tuition-waived medical education, job satisfaction, healthcare system

## Abstract

**Background:**

In order to actively cultivate a new generation of village doctors (NGVDs) who possess a high level of education, provide high-quality healthcare services, Shanghai Municipal Health Bureau, together with the Municipal Education Commission, Municipal Finance Bureau, and Municipal Agriculture Commission initiated Rural-oriented Tuition-waived Medical Education (RTME) in 2006. This study aims to identify the factors that influence the reselection intention and perceptions of NGVDs of RTME program in Shanghai.

**Methods:**

In 2023, a questionnaire survey was conducted among a sample of NGVDs enrolled in the RTME program in 8 districts of Shanghai. The research focused on five aspects, including demographic characteristics, job characteristics, reasons for choosing RTME, career identity and job satisfaction. The participants were asked questions related to their experiences, opinions, and perceptions. Analysis of variance (ANOVA), chi-square tests and logistic regression analysis was conducted.

**Results:**

A total of 419 questionnaires were collected. The majority (64.44%) indicated they would reselect this career, while 35.56% expressed either no preference or indifference. Factors influencing reselection included “Volunteering to serve the grassroots people” (odds ratio [OR], 2.29 [95% confidence interval {*CI*}, 1.22–4.30]), “Enjoying the medical profession” (OR, 2.64 [95%*CI*, 1.33–5.27]), not fulfilling family wishes (OR, 0.47 [95%*CI*, 0.24–0.88]), Satisfied with the current salary (OR, 2.39 [95%*CI*, 1.27–4.49]). NGVDs who think village doctors can leverage their professional ability with “okay” (OR, 32.87 [95%*CI*, 3.69–293.64]) and “yes” (OR, 120.03 [95%*CI*, 12.78- > 999.99]) and who think NGVDs were “important” (OR, 3.74 [95%*CI*, 1.21–11.55]) were more inclinatively to reselect to be NGVD.

**Conclusion:**

Reasons for choosing RTME, understanding of the government’s policy, career identity, and job satisfaction were all influence the reselection of RTME. This research can contribute to the improvement and development of similar programs in the future, benefiting both the students and the healthcare system as a whole.

## Background

To improve undeveloped rural health care service, Mao Zedong insisted on the need to cultivate barefoot doctors on 1965 (1, 2), who were at once amateur medical practitioners and did not have good experience in the professional medical system and were mostly recruited from ordinary villages (3, 4). Barefoot doctors were once hailed as one of the three essential weapons for rural healthcare in China by the WHO, and regarded as “a low-cost solution built around easily available indigenous medicine in the 1970s” (5). The Chinese government replaced the term “barefoot doctor” with “village doctor” in 1985 (6, 7). When passed the local examination held by the county health bureau and to obtain the Village Doctor Certification, while some village doctors could also pass the National Licensed (Assistant) Doctors Examination and become a licensed (assistant) doctor (8, 9). Barefoot doctors (BDs) and village doctors (VDs) have played an important role in providing essential and preventive health care to rural population as the most basic and extensive medical service providers in rural areas (7, 10).

Some scholars have voiced concerns that insufficient education, aging, and high turnover rates among village doctors, whose quality of the diagnostic process and outcomes was evaluated to be low (11), may limit their capacity to address the future health challenges facing the country (12, 13). In recent years, the National Health Commission of the PRC has been devoted to optimizing the team structure of village doctors and enhancing their service capabilities. In 2006, the Shanghai Municipal Health Bureau, together with the Municipal Education Commission, Municipal Finance Bureau, and Municipal Agriculture Commission, issued the “Notice on Strengthening the Training of Village Doctors in this City.” The Rural-oriented Tuition-waived Medical Education (RTME) program aims to actively cultivate a new generation of village doctors (NGVDs) who possess a high level of education, provide high-quality healthcare services, and have a younger age profile (14, 15). In this program, the medical students were going to study in designated medical school and work in designated rural township health centers. The government would waive the related tuition fee and provide them with a certain amount of living allowance (16). This is achieved through targeted enrollment, training at designated universities, directed work allocation, and strategic job placement. These initiatives ensure that the NGVDs are well-versed in rural health work, possess a certain level of general medical theory and skills, and are better equipped to serve rural areas. Shanghai University of Medicine & Health Sciences adheres to an educational philosophy that focuses on community-level healthcare, primarily training general practitioners and primary medical staff. Aligned with the Healthy China initiative and the “Healthy Shanghai” action plan, the university has successfully trained 1947 NGVDs for community-level service in recent years. These NGVDs are distributed among 117 community service centers and 223 health stations across 9 districts in Shanghai, providing fresh talent to strengthen the primary healthcare system. Today, these NGVDs who have both treatment and public health responsibilities (9) stand as the forefront of defense for community health, playing a crucial role in promoting the overall construction of a Healthy China and the rural revitalization strategy.

Researchers have recently started to focus on problems like relatively low pay, job satisfaction, disparity in human resources, social security, turnover intention, and the limited service capacity of VDs (3, 17–19). What do NGVDs, who have worked at the community level, think about the original decision to become a doctor? Do these individuals still possess the determination to choose the same path? There is a lack of in-depth literature exploring whether NGVDs have the willingness to make a new choice after reflecting on their initial decision.

To delve deeper into the question of whether NGVDs are willing to make a new choice in light of their past decisions after experiencing work at the community level, a questionnaire survey was conducted among NGVDs serving in the community. Through this survey, our objective is to analyze the perceptions of NGVDs regarding RTME, and its impact on their future intentions to work in rural areas.

## Methods

### Sampling size

Consistent with previous research findings, the turnover intention (*p*) among village doctors in China is approximately 50%, spanning a range from 35.8 to 84.44% (20–24). Employing a significance level of *α* = 0.05 for a two-tailed test, and considering a permissible margin of error (*d*) of 0.1 times the proportion (*p*), the required sample size is determined to be 400 based on the applicable statistical sampling formula: $n=\frac{Z_{n/2}^{2}\times pq}{d^{2}}$ .Considering a 10% non-response rate, the final sample size should be 440.

### Data collection

In 2023, according to the method of un-random sampling, a designed cross-sectional study was carried out and an electronic self-filled questionnaire survey was conducted among a sample of NGVDs who have worked at the community level in Shanghai. Inclusion criteria for this cross-sectional survey are as follows: Having a minimum of 6 months of clinical experience immediately preceding the survey and be consenting to engage in the survey process. The participants were asked questions related to their experiences, opinions, and perceptions. The study data were anonymous to protect privacy.

### Data measures

Based on previous studies, this study aimed to explore: (1) NGVDs reselection intentions; (2) reasons for attending RTME; (3) job satisfaction; (4) career identity; (5) the effects of job satisfaction and career on reselection intention; (6) other determinants. The questionnaire was generally divided into five parts.

- The first part, we designed relevant questions including the demographic characteristics (age, gender, education) and job characteristics (job type, working years, professional title, salary) of NGVDs.
- The second part, we asked the reasons for attending RTME with eight multiple-choice items.
- At the third part, NGVDs’ understanding of the relevant policies was measured using 2 questions, including understanding during application phase and at present.
- In the fourth part, we concerned the NGVDs’ opinion about the career identity, which three questions were collected.
- In the final part, job satisfaction (include salary, continuing education policies, competence, popular) were measured by five questions.

The NGVDs’ intention of reselecting RTME was measured by asking “If you had the chance to reselection, would you choose to be a Village Doctors of Directional Training?,” with responses of “yes,” “no” and “not clear.” The answers “yes” were coded to be yes (coded 1); while “no” and “not clear” were not (coded 0). The information collected will be kept confidential.

### Data analysis

Data analysis was performed using SAS version 9.4 and Effective results were plotted using GraphPad Prism 9. Descriptive statistics were used to describe the characteristics of the NGVDs. continuing variable such as age was presented as mean and standard deviation (SD), while the statistical description of categorical variables used frequency and percentage. Differences in “If you had the chance to choose again, would you choose to be a Village Doctors of Directional Training under the order-directed program?” were examined using ANOVA for continuing variables and *x*^2^-test for categorical variables. Finally, logistic regression analysis was conducted in order to determine factors associated with the intention of NGVDs reselect RTME. A *p*-value =0.05 was considered significant.

## Results

### Basic information

A total of 419 participants were successfully recruited, although 21 participants did not complete the questionnaire in accordance with the specified requirements. The NGVDs’ average age were 27.31 ± 2.59 years, with 25.06% male and 74.94% female respondents. In terms of the respondents’ work unit levels, 21.96% were from township health centers, while the majority, 76.61% of the NGVDs, were from community health service centers. 98.09% were Still practicing as a doctor, with the rest worked in other types of jobs. Among the respondents, 49.40% of which had less than 3 years of work experience, only 1.7% of them held medium-grade and above professional titles (see Table 1).

**Table 1:** NGVDs’ demographics (*n* = 419).

Variables	Category	N/Mean ± SD	Percentage %
Age		27.31 ± 2.59	
Gender	Male	105	25.06
	Female	314	74.94
Education	Junior college	165	39.38
	Bachelor’s degree and above	254	60.62
Current workplace	Township health center	92	21.96
	Community health service center	321	76.61
	Tertiary hospital and other	6	1.44
Job type	Physician	411	98.09
	Administrative management	2	0.48
	Other	6	1.43
Years of working	≤3	207	49.40
	3–6	150	35.80
	>6	61	14.56
Professional title	Primary	412	98.33
	Medium-grade and above	7	1.67
Current monthly salary? (Yuan)	3,000–4,500	118	28.16
	4,500–6,000	147	35.08
	6,000–8,000	117	27.92
	8,000–10,000	30	7.16
	>10,000	7	1.67

### Reasons for applying as village doctors of directional training

When asked about the reasons for applying as NGVD of RTME, the top three were “No job pressure as work unit determined upon enrollment,” “Volunteering to serve the grassroots people” and “No financial pressure during schooling period” (see Table 2).

**Table 2:** The reasons for applying for village doctors of RTME.

What was your reason for applying for the directed free medical student program at that time?	*N*	Percentage %
No job pressure as work unit determined upon enrollment	296	70.64
Volunteering to serve the grassroots people	129	30.79
No financial pressure during schooling period	122	29.12
Enjoying the medical profession	101	24.11
Hoping to work in hometown after graduation	98	23.39
Fulfilling family wishes	71	16.95
Level of scores determining	59	14.08
Others	30	7.16

### Temporal changes

During the initial application phase, 16.23% of them had a thorough understanding of the government’s policy on RTME, while 47.26% had some understanding. 29.83% had a general understanding, and 6.68% had no understanding of the policy. In the current stage, these proportions have changed to 18.62, 50.12, 26.73, and 4.54%, respectively.

**Figure 1:**
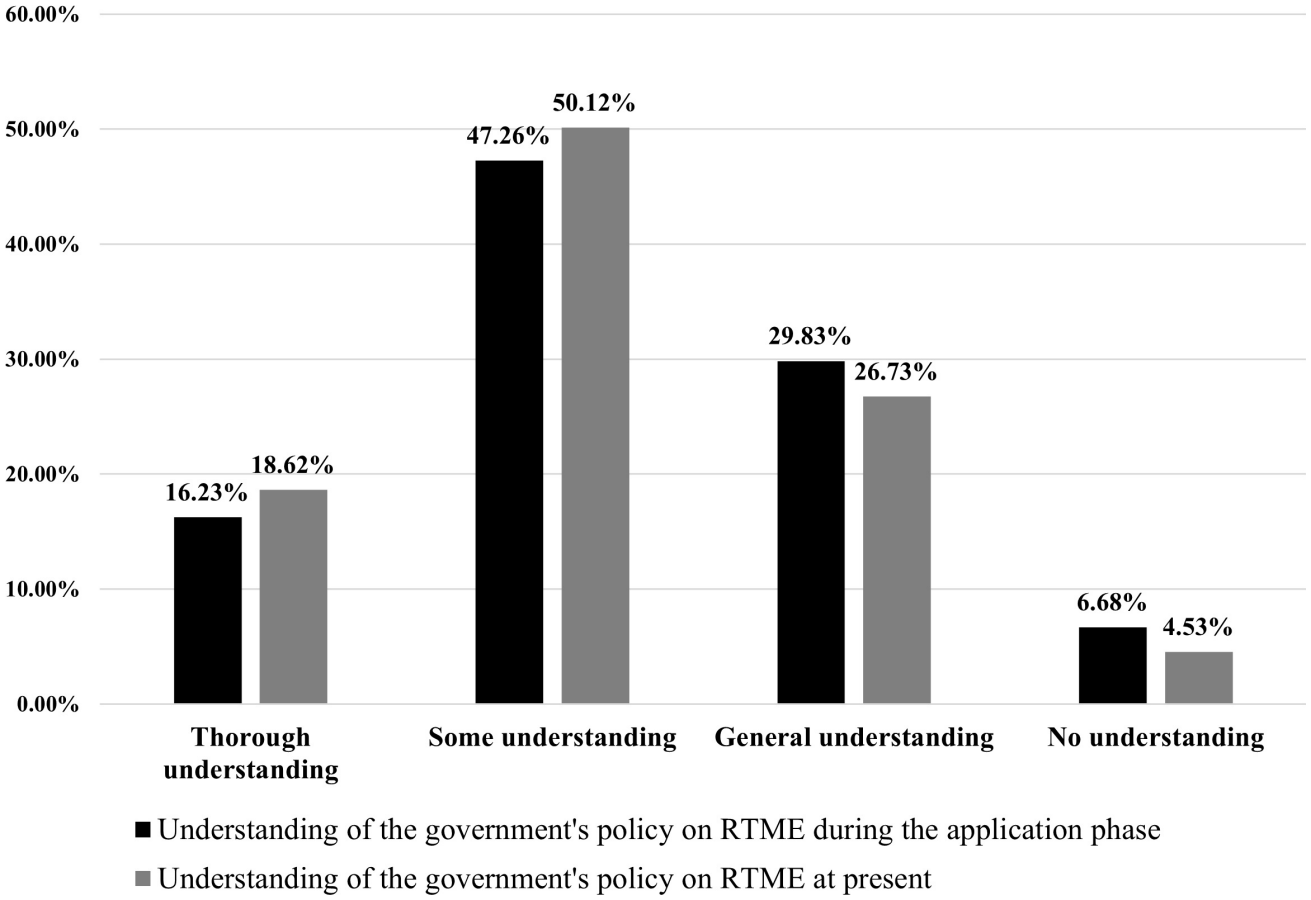
The comparison of policy understanding between the past and present.

Based on the data in Figure 1, there has been a significant increase in the level of understanding of the government’s policy on RTME compared to the application phase. Initially, 63.49% of individuals had a high level of understanding of the policy. In the current stage, this proportion has increased to 68.74%, representing a 5.25% increase compared to the application phase. Conversely, during the application phase, 36.51% of individuals did not have a deep understanding of the policy. In the current stage, only 31.24% of individuals lack a deep understanding, indicating a 5.17% decrease compared to the application phase.

**Figure 2:**
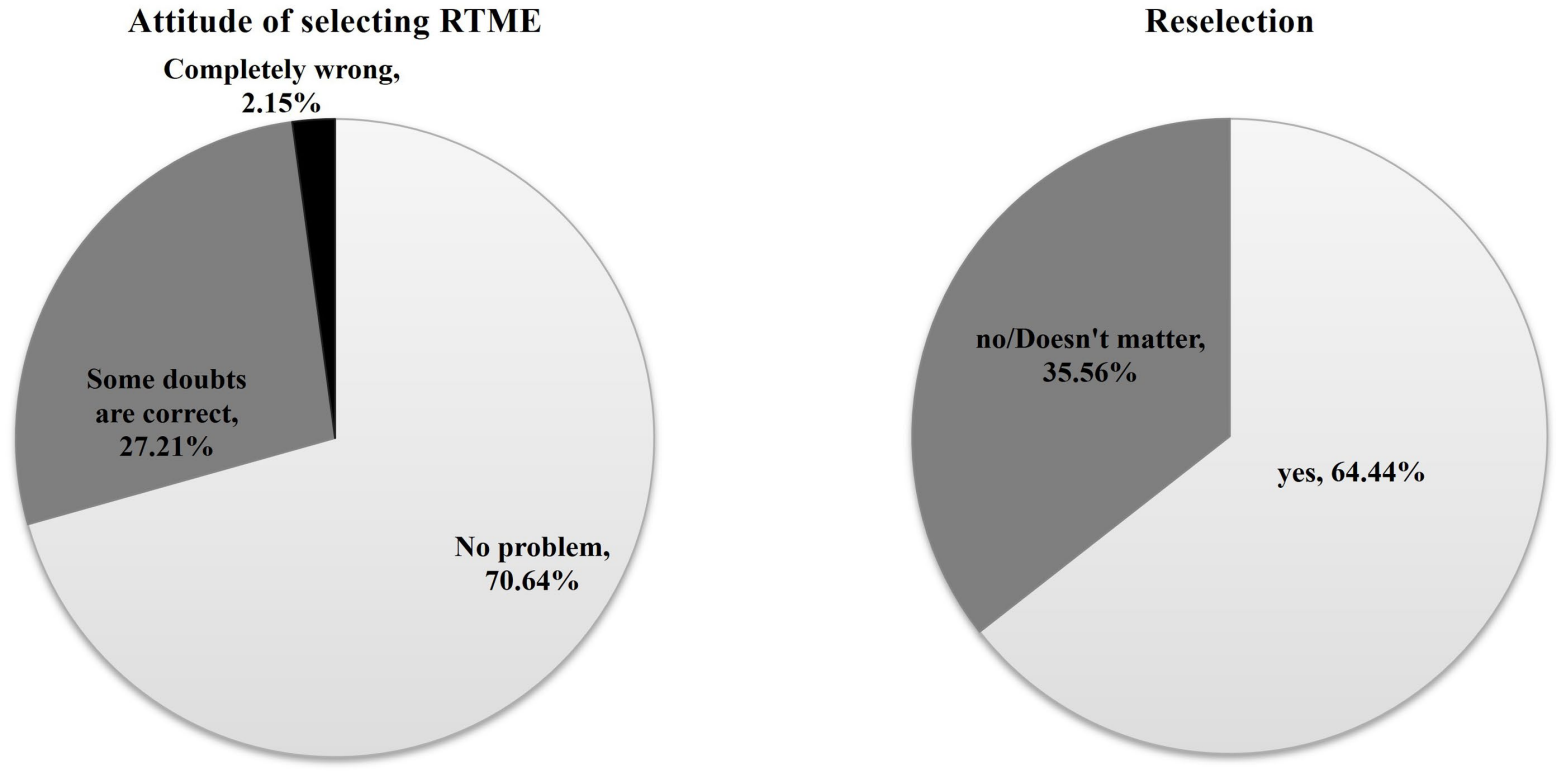
Attitude of selecting and reselection of RTME.

In terms of how they view the signing of the RTME, 70.64% believe that their initial choice was correct. On the other hand, 27.21% express doubt about their initial choice, and 2.15% admit that their initial choice was completely wrong (see Figure 2).

### Factor associated with reselection

In the case of reselection, 270 individuals (64.44%) expressed their willingness to select the RTME again, while 149 individuals (35.56%) indicated that they would not (no clear) select it again.

From Table 3, it shows that the reasons for reselecting RTME such as “Volunteering for grassroots service” and “enjoying the medical profession” and “meeting family expectations” were related to the intention to reselection (*p* < 0.05). In the face of reselection, different choices will be made based on these reasons. The level of understanding of the national policy during two stages, importance of VDs and job satisfaction were all related to the intention to reselection (*p* < 0.05).

**Table 3:** Factors influencing reselection of NGVDs in the RTME program.

	If you had the chance to reselection, would you choose to be a village doctors of directional training?			*t/χ* ^2^	*P*
Variables	Category	Yes	No		
		(N = 270)	(N = 149)		
Age		27.37 ± 2.53	27.20 ± 2.69	0.64	0.52
Gender	Male	69 (65.17%)	36 (34.29%)	0.10	0.75
	Female	201 (64.01%)	113 (35.99%)		
Education	Junior college	105 (63.64%)	60 (36.36%)	0.08	0.78
	Bachelor’s degree and above	165 (64.96%)	89 (35.04%)		
Professional title	Primary	266 (64.56%)	146 (35.44%)	0.17	0.68
	Medium-grade and above	4 (57.14%)	3 (42.86%)		
Current monthly salary? (Yuan)	3,000–4,500	62 (52.54%)^a^	56 (47.46%)	12.69*	0.013
	4,500-6000	97 (65.99%)^a,b^	50 (34.01%)		
	6,000–8,000	87 (74.36%)^b^	30 (25.64%)		
	8,000–10,000	20 (66.67%)^a,b^	10 (33.33%)		
	>10,000	4 (57.14%)^a,b^	3 (42.86%)		
RTME for volunteering to serve the grassroots people	No	167 (57.59%)	123 (42.41%)	19.30	<0.01
	Yes	103 (79.84%)	26 (20.16%)		
RTME for enjoying the medical profession	No	188 (59.12%)	130 (40.88%)	16.29	<0.01
	Yes	82 (81.89%)	19 (18.81%)		
RTME for fulfilling family wishes	No	233 (66.95%)	115 (33.05%)	5.67	0.017
	Yes	37 (52.11%)	34 (47.89%)		
Understanding of the government’s policy on RTME during the application phase	Thorough understanding	61 (89.71%)^a^	7 (10.29%)	32.28*	<0.01
	Some understanding	131 (66.16%)^b^	67 (33.84%)		
	General understanding	66 (52.80%)^b^	59 (47.20%)		
	No understanding	12 (42.86%)^b^	16 (54.14%)		
Understanding of the government’s policy on RTME at present	Thorough understanding	66 (84.62%)^a^	12 (15.38%)	31.79*	<0.01
	Some understanding	142 (67.62%)^b^	68 (32.38%)		
	General understanding	52 (46.43%)^c^	60 (53.57%)		
	No understanding	10 (52.63%)^b,c^	9 (47.37%)		
What is your opinion on the impact of primary health care on career development?	Positive impact, plays a promotive role in career development	214 (75.35%)^a^	70 (24.65%)	59.35	<0.01
	Hinders career development	7 (31.88%)^b^	25 (78.13%)		
	No impact	22 (64.71%)^a,c^	12 (35.29%)		
	Unclear	27 (39.13%)^b,c^	42 (60.87%)		
NGVDs can leverage their professional ability?	Yes	173 (83.98%)^a^	33 (16.02%)	86.62*	<0.01
	Okay	96 (50.53%)^b^	94 (49.47%)		
	No	1 (4.35%)^c^	22 (95.65%)		
Importance of NGCDs assigned to primary health care development	Important	258 (70.11%)^a^	110 (29.89%)	42.42*	<0.01
	Not very/Important	7 (24.14%)^b^	22 (75.86%)		
	Unfamiliar	5 (22.73%)^b^	17 (77.27%)		
Satisfaction with the current salary	Satisfied	97 (82.20%)	21 (17.80%)	22.62	<0.01
	Dissatisfied	173 (57.48%)	128 (42.52%)		
Satisfaction with the performance of professional competence	Very satisfied	65 (84.42%)^a^	12 (15.58%)	21.17*	<0.01
	Generally satisfied	199 (61.23%)^b^	126 (38.77%)		
	Dissatisfied	6 (35.29%)^b^	11 (64.71%)		
Satisfaction with the continuing education policies	Very satisfied	88 (79.28%)^a^	23 (20.72%)	17.03*	<0.01
	Generally satisfied	175 (60.14%)^b^	116 (39.86%)		
	Dissatisfied	7 (41.18%)^b^	10 (58.82%)		
Popularity at your workplace	Very popular	33 (76.74%)^a^	10 (23.26%)	31.42*	<0.01
	Popular	227 (67.56%)^a^	109 (32.44%)		
	Not popular (Excluded)	10 (25.00%)^b^	30 (75.00%)		
Current salary affects the reselection	No	51 (85.00%)	9 (15.00%)	12.92	<0.01
	Yes	219 (61.00%)	140 (39.00%)		

In the same column, letters a, b, c denote that different letters indicate statistically significant differences between different rows. *Tested by the Cochran-Armitage trend, *p* < 0.001.

Variables that achieve statistical significance in the t-test or chi-square test are incorporated into the logistic regression analysis. Table 4 shows the results of the logistic regression for factors associated with reselection. NGVDs who chose the reason for “Volunteering to serve the grassroots people” (*p* < 0.05, OR = 2.29, 95%*CI*: 1.22–4.30), “Enjoying the medical profession” (*p* < 0.05, OR = 2.64, 95%*CI*: 1.33–5.27) and not fulfilling family wishes (*p* < 0.05, OR = 0.47, 95%*CI*: 0.24–0.88) were more likely to reselect this career. NGVDs who think village doctors can leverage their professional ability with “okay” (*p* < 0.05, OR = 32.87, 95%*CI*: 3.69–293.64) and “yes” (*p* < 0.05, OR = 120.03, 95%*CI*: 12.78- > 999.99) and who think NGVDs were “important” (*p* < 0.05, OR = 3.74, 95%*CI*: 1.21–11.55) were more inclinatively to reselect to be NGVD. Satisfied with the current salary also increased their probability of reselecting this profession (*p* < 0.05, OR = 2.39, 95%*CI*: 1.27–4.49).

**Table 4:** Logistic regression analysis of factors influencing reselection of NGVDs in the RTME program.

Variables	Category	Crude OR(95%*CI*)	Adjusted OR(95%*CI*)*	*χ* ^2^	*P*
Gender	Male	1 [Reference]	1 [Reference]		
	Female	0.93 (0.58–1.48)	0.85 (0.47–1.54)	0.30	0.58
Education	Junior college	1 [Reference]	1 [Reference]		
	Bachelor’s Degree and above	1.06 (0.7–1.59)	1.37 (0.82–2.31)	1.43	0.23
Professional title	Primary	1 [Reference]	1 [Reference]		
	Medium-grade and above	0.73 (0.16–3.31)	0.99 (0.1–9.49)	0.00	0.99
RTME for volunteering to serve the grassroots people	No	1 [Reference]	1 [Reference]		
	Yes	2.92 (1.79–4.76)	2.29 (1.22–4.30)	6.58	0.01
RTME for enjoying the medical profession	No	1 [Reference]	1 [Reference]		
	Yes	2.98 (1.73–5.16)	2.64 (1.33–5.27)	7.62	0.01
RTME for fulfilling family wishes	No	1 [Reference]	1 [Reference]		
	Yes	0.54 (0.32–0.9)	0.47 (0.24–0.88)	5.46	0.02
Understanding of the government’s policy on RTME at present?	Thorough understanding	1 [Reference]	1 [Reference]		
	Some understanding	0.38 (0.19–0.75)	0.66 (0.27–1.61)	0.82	0.36
	General understanding	0.16 (0.08–0.32)	0.34 (0.13–0.87)	5.05	0.02
	No understanding	0.2 (0.07–0.6)	1.17 (0.25–5.45)	0.04	0.84
NGVDs can leverage their professional ability?	No	1 [Reference]	1 [Reference]		
	Okay	22.44 (2.97–169.70)	32.87 (3.69–293.64)	9.77	0.00
	Yes	115.20 (15.02–883.56)	120.03 (12.78- > 999.99)	17.55	<0.0001
Importance of NGVDs assigned to primary health care development:	Not very/Important	1 [Reference]	1 [Reference]		
	Important	7.37 (3.06–17.76)	3.74 (1.21–11.55)	5.25	0.02
	Unfamiliar	0.92 (0.25–3.43)	1.20 (0.26–5.59)	0.05	0.82
Satisfaction with the current salary	Dissatisfied	1 [Reference]	1 [Reference]		
	Satisfied	3.42 (2.02–5.77)	2.39 (1.27–4.49)	7.34	0.01
Satisfaction with the performance of professional competence	Very satisfied	1 [Reference]	1 [Reference]		
	Generally satisfied	0.29 (0.15–0.56)	1.51 (0.62–3.67)	0.82	0.37
	Dissatisfied	0.1 (0.03–0.32)	3.87 (0.7–21.55)	2.39	0.12
Current salary affects the reselection	No	1 [Reference]	1 [Reference]		
	Yes	0.28 (0.13–0.58)	0.39 (0.14–1.07)	3.36	0.07

^*^Adjusted for gender, education, professional title.

## Discussion

### Reselection

Numerous similar studies have made research on turnover intention which refers the likelihood of an employee to leave the current job he/she are doing (25). In our study, 35.56% of the NGVDs will not reselect RTME, while the rest of them will still reselect this career which is lower than the rate of other provinces in China. 66.5% of rural-oriented tuition-waived medical students (RTMSs) in Shaanxi had no intention to remain after the contract expired (20) while 57.7% RTMSs in Shandong province, 84.44% in Chongqing and 45.5% in Chengdu, had low and medium willingness to fulfill the contract (21). A investigation results showed that 36.8% of the village doctors in Xiangyang City intended to resign (22) and the number was 42.3% in Chongqing (23). A survey about village doctor in Shandong province showed that 46.9% of them had a higher turnover intention (24). According to systematic research, which integrated 20 research among 23,284 VDs in China, the prevalence of turnover intention among VDs in China was as high as 44.1% (3). A study of comparison based on three surveys in a province in eastern China found that the turnover intention of village clinic doctors has increased by time (26).

But the rate is higher than Inner Mongolia autonomous region which approximately 33% of rural order-oriented medical students were not willing to work in rural areas (27). The other counties, Grassroots doctors in British, for example, only 11.8% of primary care family physicians had high turnover intention (28). Of a 1,174 family physicians studied in England, only 6.30% of them stated high intention to leave (29).

### Career identity

This study showed clearly the top three reasons for applying as NGVD of RTME were guaranteed employment, volunteering to serve the grassroots people and no financial pressure, which is in accordance with findings in previous studies (16). Understanding the motivation why they select RTME program is very important because a motivated individual is willing to exert and maintain an effort to provide good quality health services (30). Simultaneously, we reported that students who enrolled in the RTME program due to “Volunteering to serve the grassroots people” and “Enjoying the medical profession” were more likely to reselect this career. But, “RTME for Fulfilling family wishes” were less likely to reselect the RTME program. Therefore, medical schools and employers should focus on researching how to correctly improve the professional identity of village doctors, so that the RTME project can sustainably develop and achieve maximum resource utilization.

The results of our study showed that career identity related to reselection conclude “NGVDs can leverage their professional ability” and “Importance of NGVDs assigned to primary health care development.” Study by Zhang et al. strongly confirmed that career identity are early and powerful predictors of the willingness to fulfill the contract of RTMSs (21). Village doctors had poor working and living conditions, such as low income, lack of social security, inappropriate performance assessment, inadequate training, heavy workload and insufficient cooperation from rural residents (31), which affected the career identity of them (32). Simultaneously, limited professional development prospect, unsound medical equipment and facilities, and inadequate financial remuneration, influencing the professional identity of RTMSs (33).

### Job satisfaction

Job satisfaction is an important predictor and it have a direct and indirect negative effect on turnover intention of health workers (24, 34). A scientometrics perspective study by Chen highlighted that job satisfaction had a negative predictive effect on VDs’ turnover intention (35). Our study indicated that NGVDs were dissatisfied with their jobs, including salary and performance of professional competence and continuing education policies, especially among those who will not reselect NGVD of RTME. A cross-sectional study showed 48.6% of VDs felt satisfied with their job (36). Renmin Jin’s study found that job satisfaction rate of village doctors with RTME program was 56.50% (16).

Studies showed that income satisfaction contribute to the turnover intention of village doctors (22, 37). Income can be a major contributor to dissatisfaction and low income was directly linked to the retention rates of rural physicians (38). The mismatch between income and workloads is a major contributor toward job dissatisfaction (39). This result showed that among the 419 NGVDs surveyed, more than half of them, 301 (71.84%) dissatisfied with the current salary. Huang’s survey of village doctors’ mobility found that 70% of village doctors were unsatisfied with their income (40). A series of changes, such as cancelling drug mark-ups and integrating management of village doctors’ activities, have dramatically reduced village doctors’ income and employment satisfaction (41).

### Healthy policy

The research findings suggest that a considerable number of NGVDs are inclined to opt for the RTME program again, indicative of its favorable reception. Nonetheless, delving into the motivations behind the reluctance of others is imperative for refining enrollment and retention approaches. Although the RTME program has played a pivotal role in mitigating the scarcity of healthcare personnel in rural regions, the study underscores key domains that necessitate policy interventions to bolster the program’s impact and long-term viability.

The study suggests that limited professional development prospects and financial remuneration affect the professional identity of NGVDs. Health policies should focus on fostering a strong professional identity among medical students by emphasizing the importance of their role in rural healthcare and providing opportunities for community engagement. Moreover, policies must be crafted to present enduring career pathways, which include prospects for specialization and continuing education within the rural healthcare infrastructure. Given the extensive training requirements of village doctors (42), it is imperative to develop a standardized Continuing Medical Education (CME) framework. This framework should guarantee NGVDs access to ongoing training in both clinical and public health domains, thereby enhancing their skills and knowledge, and ultimately elevating the quality of care they deliver.

Consistent with prior empirical research that corroborated the direct association between job satisfaction and the propensity for turnover (43), the current investigation reveals that job satisfaction, specifically related to salary and career advancement opportunities, is a crucial determinant in the reselection decision-making process of NGVDs. Health policies should aim to improve the compensation and benefits for NGVDs. Tackling the challenges of substandard income and insufficient social protection is imperative for the recruitment and retention of skilled NGVDs. The adoption of policies guaranteeing equitable compensation and benefits, including performance-related pay and extensive insurance schemes, is likely to bolster job satisfaction and enhance staff retention rates.

This investigation underscores the critical role that workplace conditions and support systems play in affecting the reselection of NGVDs. Health policies must prioritize the enhancement of the work environment, the provision of sufficient resources, and the establishment of social security measures for these healthcare professionals. Additionally, health policies could foster community engagement in healthcare provision and establish support networks for NGVDs, thereby reinforcing their sense of belonging and societal contribution. Establishing peer support networks, which appear to substantially impact job satisfaction (44), can offer NGVDs a communal and emotional lifeline. Ensuring access to mental health support services, including counseling and stress management initiatives, is vital for helping them navigate the emotional and psychological demands of rural healthcare work.

### Limitation

This study has several limitations. First, data was collected using convenience sampling, not representative of the experience of all NGVDs in Shanghai. Meanwhile our study provides valuable insights into the reselection intentions of NGVDs in Shanghai, it is important to acknowledge the potential limitations in the generalizability of our findings. The sample was drawn from a specific urban setting in Shanghai, which may not fully represent the experiences and perspectives of NGVDs in rural or less developed areas. Second, the cross-sectional study design made it difficult to identify causal relationships. The cross-sectional nature of our study precludes the examination of changes over time which means that we cannot track the development of phenomena or assess the stability of the observed associations. Further study with a longitudinal design is needed. Third, data was collected by self-report of the respondents, which could cause recall bias, and structured scales were not used to measure turnover intention, job satisfaction, and career identity. These limitations should be considered in future studies.

### Result generalizability

While acknowledging the limitations inherent in our study’s design and sample, several aspects of our findings suggest potential broader applicability beyond the specific context of Shanghai. The identified factors influencing reselection intentions, namely career identity, job satisfaction, and understanding of government policies, are likely to be relevant in other settings where rural-oriented tuition-waived medical education programs are implemented. Furthermore, the challenges faced by VDs, including low income and limited professional development opportunities, are widely recognized issues impacting healthcare workers in rural areas across China.

## Conclusion

The results highlight the importance of teaching quality, internship experiences, reasons for choosing the program, understanding of government policy, promotion determinants, salary levels, and workplace popularity. First, measures such as classroom education and early clinical exposure were taken to strengthen the professional identity of medical students. Second, authorities are expected to offer supportive policies, especially in terms of professional title assessment, continuing education and income. Our study offers cues for health care policy maker and educators to implement a broader national process and organizational strategies to improve the job satisfaction and career identity of the NGVDs.

## Data Availability

The raw data supporting the conclusions of this article will be made available by the authors, without undue reservation.
